# Harvard Medical School

**Published:** 1863-04

**Authors:** 


*Harvard Medical School.*—At the annual commencement, on the 11th of March, the degree of M. D. was conferred on 42 candidates.

